# Met and Unmet Needs of Cognitively Impaired Older Adults and Burden and Benefits of Their Caregivers

**DOI:** 10.1093/geroni/igab046.1077

**Published:** 2021-12-17

**Authors:** Pildoo Sung, Johan Suen, Nawal Hashim, Rahul Malhotra, Angelique Chan

**Affiliations:** Duke-NUS Medical School, Singapore, Singapore

## Abstract

Previous studies typically assess caregiver needs when trying to interpret caregiver burden. We propose that both met and unmet needs of care recipients translate into different caregiving experiences with varying levels of benefits and burden combined. We use data on 263 caregivers of community-dwelling Singaporean older adults with cognitive impairment who participated in a community-based dementia care study conducted in 2018-2020. Our analysis produces three major findings. First, latent class analysis identifies three distinct types of caregiving experience based on caregiver-reported burden and benefits of caregiving: intensive (high burden and high benefits, 11% of caregivers), satisfied (low burden and high benefits; 54%), and dissatisfied (low burden and low benefits; 35%). Second, multinomial logistic regression shows that both met and unmet needs of care recipients are positively associated with the intensive caregiving experience, while only met needs are positively associated with the satisfied caregiving experience, in comparison to dissatisfied caregiving experience. Third, met needs in the areas of daytime activities, memory assistance, and mobility are positively related to the satisfied caregiving experience, compared to the dissatisfied caregiving experience. In other words, caregivers are more likely to be satisfied in their caregiving experiences (i.e., low burden and high benefits) if their care recipients’ problems with memory, mobility, and finding suitable and adequate daytime activities are properly managed. Our findings thus call for interventions to fulfill care recipients’ needs in a more tailored manner in order to increase satisfaction among caregivers.

